# Cancer‐related stroke: Exploring personalized therapy strategies

**DOI:** 10.1002/brb3.2738

**Published:** 2022-08-08

**Authors:** Yu‐Jie Chen, Rui‐Guo Dong, Meng‐Meng Zhang, Chao Sheng, Peng‐Fei Guo, Jie Sun

**Affiliations:** ^1^ Department of Neurological Rehabilitation Xuzhou Central Hospital Xuzhou city P.R. China; ^2^ Department of Neurology The Affiliated Hospital of Xuzhou Medical University Xuzhou city P.R. China

**Keywords:** cancer‐related stroke, embolic stroke of undetermined source, hypercoagulability, individualized therapy, pathogenesis, stroke subtype

## Abstract

**Background:**

Cancer and ischemic stroke are two common diseases that threaten human health and have become the main causes of death in the world. It is estimated that one‐in‐ten patients with ischemic stroke have concomitant cancer, and this incidence is expected to increase as improvements in medical technology extends the life expectancy of cancer patients.

**Discussion:**

Cancer‐related stroke (CRS) refers to unexplained ischemic stroke in patients with active cancer that cannot be explained by current stroke mechanisms. Available evidence suggests that CRS accounts for 5–10% of embolic stroke of undetermined source (ESUS). Although the incidence of CRS is gradually increasing, its underlying pathogenesis remains unclear. Also, there is no consensus on acute treatment and secondary prevention of stroke.

**Conclusion:**

In this review, we retrospectively analyzed the incidence, mechanisms of CRS, its potential as a new stroke subtype, options for acute treatment, secondary prevention strategies, and disease progression, with the aim of attempting to explore personalized therapy strategies.

## Graphical abstract

We discussed the possible pathogenesis of cancer‐related stroke and explored tailored treatment options based on differences in pathogenesis.

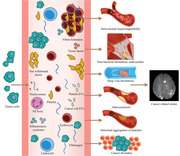

## 1 INTRODUCTION

Cancer and stroke are common diseases that are currently threatening human health. Evidence has shown that the risk of ischemic stroke begins to increase in the early stages of cancer diagnosis (Jang et al., 2019; Navi et al., 2018, 2015, 2018, 2017, 2019). In a health insurance‐based study, 6.9% of elderly lung cancer patients had an ischemic stroke one year after cancer diagnosis, versus 3.2% in a matched control group (Navi et al., 2017). With the aging population worldwide (Tu et al., 2022) and the advancement of medical treatment standards, the incidence of cancer‐related stroke (CRS) is expected to increase (Strongman et al., 2019). One‐quarter to one‐third of ischemic strokes have no established mechanism after standard diagnostic evaluation and are classified as embolic stroke of undetermined source (ESUS). CRS is a unique and important subgroup in ESUS, accounting for 5–10% of ESUS. Compared with traditional stroke, CRS is unique in clinical characteristic, underlying pathophysiologies, and treatment and prognostics, and is anticipated to be as common as traditional stroke subtypes such as large atherosclerosis, small artery occlusion, and cardiac embolism. Recently, Bang et al. (2020) and Navi & Iadecola (2018) proposed that CRS is an emerging subtype of ischemic stroke, with many potential mechanisms for the occurrence of heterogeneity. Of these mechanisms, cancer‐related hypercoagulability (CRH) is probably the most important. Therefore, CRS may be treated with anticoagulation therapy theoretically. However, the neutral result of TEACH pilot trial (Navi, Marshall, et al., 2018) suggested that compared with antiplatelet therapy, anticoagulation did not show obvious advantages in preventing stroke recurrence and bleeding. Unfortunately, the results of other clinical trials of anticoagulant therapy in CRS patients are not very optimistic, either (Nam, Kim, Kim, An, Oh, et al., 2017; Yamaura et al., 2021). Therefore, the effectiveness of anticoagulation therapy needs to be evaluated further. In the future, it is necessary to deeply explore the underlying pathophysiological mechanism of this disease and formulate unified management practices. In this article, we will discuss the possibility of CRS as a stroke subtype, review the latest developments in the mechanism of CRS and related treatment strategies, and try to summarize how to devise current treatment plans based on individual pathogenic mechanisms in Table 1. We hope to provide references for the formulation of personalized therapy strategies.

**TABLE 1:** Exploration of individualized therapy based on underlying mechanisms

Mechanisms where anticoagulation therapy may be effective	Possible reasons	Alternative treatment
Intravascular coagulopathy	TF activation, elevated thrombin; elevated D‐dimer	DOAC, heparin anticoagulant
NBTE	Intravascular coagulopathy, endothelial damage	Heparin anticoagulant, heart valve surgery
Paradoxical embolism	Intravascular coagulopathy, VTE, PFO	DOAC, heparin anticoagulant, foramen ovale closure
**Mechanisms where anticoagulation therapy may be ineffective**		
Atherosclerosis	Vascular plaque, vascular injury	Antiplatelet, thrombolysis and thrombectomy (cannot be excluded), avoid offending radiotherapy
Abnormal platelet aggregation	Elevated platelet activation markers	Antiplatelet, surgical resection, chemotherapy or radiotherapy
Cancer thrombus and cancer comorbidities	Cancer thrombus, infection, radiotherapy damage blood vessels	Surgical resection, avoid offending radiotherapy, comprehensive care

Abbreviations: TF, tissue factor; DOAC, direct oral anticoagulants; NBTE, non‐bacterial thrombotic endocarditis; VTE, venous thromboembolism; PFO, patent foramen ovale.

## 2 CRS CHALLENGES TOAST SUBTYPES

**FIGURE 1:**
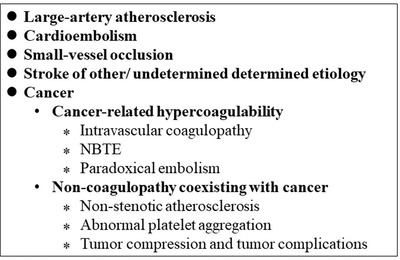
CRS challenges TOAST subtypes

Stroke can be caused by different reasons, which can affect the prognosis, outcome, and management of each case. The etiology classification based on the various causes of stroke, namely the TOAST classification (Adams et al., 1993), is currently the most widely used stroke classification system. The TOAST classification etiologically divides the causes of strokes into five possibilities: (i) large atherosclerosis, (ii) small vessel occlusion, (iii) cardiogenic, (iv) other causes, and (v) unexplained types. In this classification system, cancer is not mentioned as a potential cause. However, with the increase in the incidence of CRS and the research progress on the pathogenesis of this stroke, the existing arrangement may not meet the needs of today's clinical medicine. Therefore, a more complete classification of the etiology of stroke is needed to facilitate faster selection of appropriate treatment methods for patients and improve their prognosis (Figure 1). It has been mentioned in the TOAST trial that if a factor is used as a unique cause of stroke, it should be based on the risk factors, clinical characteristics (laboratory, imaging), prognosis, and treatments that are linked to the cause.

Growing data suggests a potential relationship between stroke and cancer. A national study in Sweden found that the overall risks of patients developing ischemic and hemorrhagic stroke within 6 months of cancer diagnosis were 2.2 (95% confidence interval [CI] = 2.0–2.3) and 1.6 (CI = 1.5–1.6), respectively (Zöller et al., 2012). Previous studies have shown that approximately 4–12% of patients with ischemic stroke have active cancer (Gon et al., 2016; Grazioli et al., 2018) and that the link may be on the rise. Among different types of the disease, lung cancer and digestive system cancer are the ones that are more closely related to the incidence of stroke (Chen et al., 2015; Kato et al., 2016; Lin et al., 2019; Navi, Howard, et al., 2018). It was found in a large population‐based cohort that the risk of ischemic stroke began to increase 5 months before cancer diagnosis, suggesting that some cryptogenic stroke may be caused by occult cancer (Navi et al., 2019). Cancer is often underestimated as a potential risk factor for stroke, so some CRS patients are often discovered at autopsy. It is reported in an autopsy study that 15% of cancer patients have cerebrovascular disease (Rogers, 2010). These evidences all indicate that cancer is a risk of stroke.

When CRS occurs, it is often accompanied by unique clinical features. In the laboratory, it is manifested as hypercoagulability (el‐Shami et al., 2007; S. G. Kim et al., 2010; Seok et al., 2010). In imaging, it is manifested as multiple infarcts on the diffusion‐weighted image (DWI) involving multiple vascular areas. On magnetic resonance image (MRI), CRS often appears as a ring‐shaped area of ​​restricted diffusion with a diameter of 0.5–2 cm, which is mainly located in areas with ​​large blood vessels and usually does not accumulate cortex or deep gray matter (Finelli & Nouh, 2016). Existing evidence suggests that multivessel artery infarction and elevated D‐dimer levels are independent predictors of stroke associated with malignant tumors (S. G. Kim et al., 2010; S. J. Kim et al., 2012; Wang et al., 2018).

Studies have shown that patients with cancer complicated with ischemic stroke have a high risk of short‐term stroke recurrence and a poor prognosis. These surveys found that in patients diagnosed with cancer, the cumulative recurrence rate of stroke 1 month after the first episode is 7% (Chow et al., 2018; Navi et al., 2014), and the recurrence rate at 6 months reached 16% (Soda et al., 2004). Patients with malignant tumors also have a higher risk of venous and arterial embolism, and if cancer patients develop these clinical conditions after a stroke, they can have a significant negative impact on the patient's 1‐year survival rate (Ha et al., 2019). Due to the unclear pathogenesis and lack of effective secondary prevention strategies, even with the use of anticoagulants, 17.2% of patients with active cancer experienced a recurrence of stroke during a median observation period of 62 days after the first stroke occurred (Ohara et al., 2020). So patients with CRS usually have a poor prognosis and a higher risk of death. In a survey involving 14,358 tumor participants, the follow‐up analysis pointed out that 224 patients had a stroke after being diagnosed. Among the 2636 deaths, the all‐cause mortality rate without stroke was 0.70(95% CI, 0.68∼0.73). The mortality rate after the first stroke was 1.03(95% CI, 0.73–1.46), and the all‐cause mortality rate after stroke recurrence was 2.42(95% CI, 1.48–3.94) (Nam, Kim, Kim, An, Demchuk, et al., 2017).

A recent prospective study of 50 CRS patients found that these patients have higher coagulation, platelet, and endothelial dysfunction markers, and more circulating micro‐emboli. Compared with patients with only acute ischemic stroke or only active cancer, these differences are statistically significant. The biological markers found in this study (D‐dimer, thrombin‐antithrombin, P‐selectin, thrombomodulin, soluble intercellular adhesion molecule‐1[sICAM‐1], soluble vascular cell adhesion molecule‐1[sVCAM‐1]) has high practicability in clinical practice and is of great significance in predicting the occurrence of CRS. This can also improve our understanding of the mechanism of ischemic stroke in cancer patients. In this context, the occurrence of stroke is considered to be closely related to hypercoagulability (Navi et al., 2021). Therefore, different from the pathogenesis of traditional cerebral infarction, the choice of antithrombotic drugs for this type of patient may differ from traditional options.

## 3 MECHANISMS WHERE ANTICOAGULATION THERAPY MAY BE EFFECTIVE

### 3.1 Intravascular hypercoagulability

**FIGURE 2:**
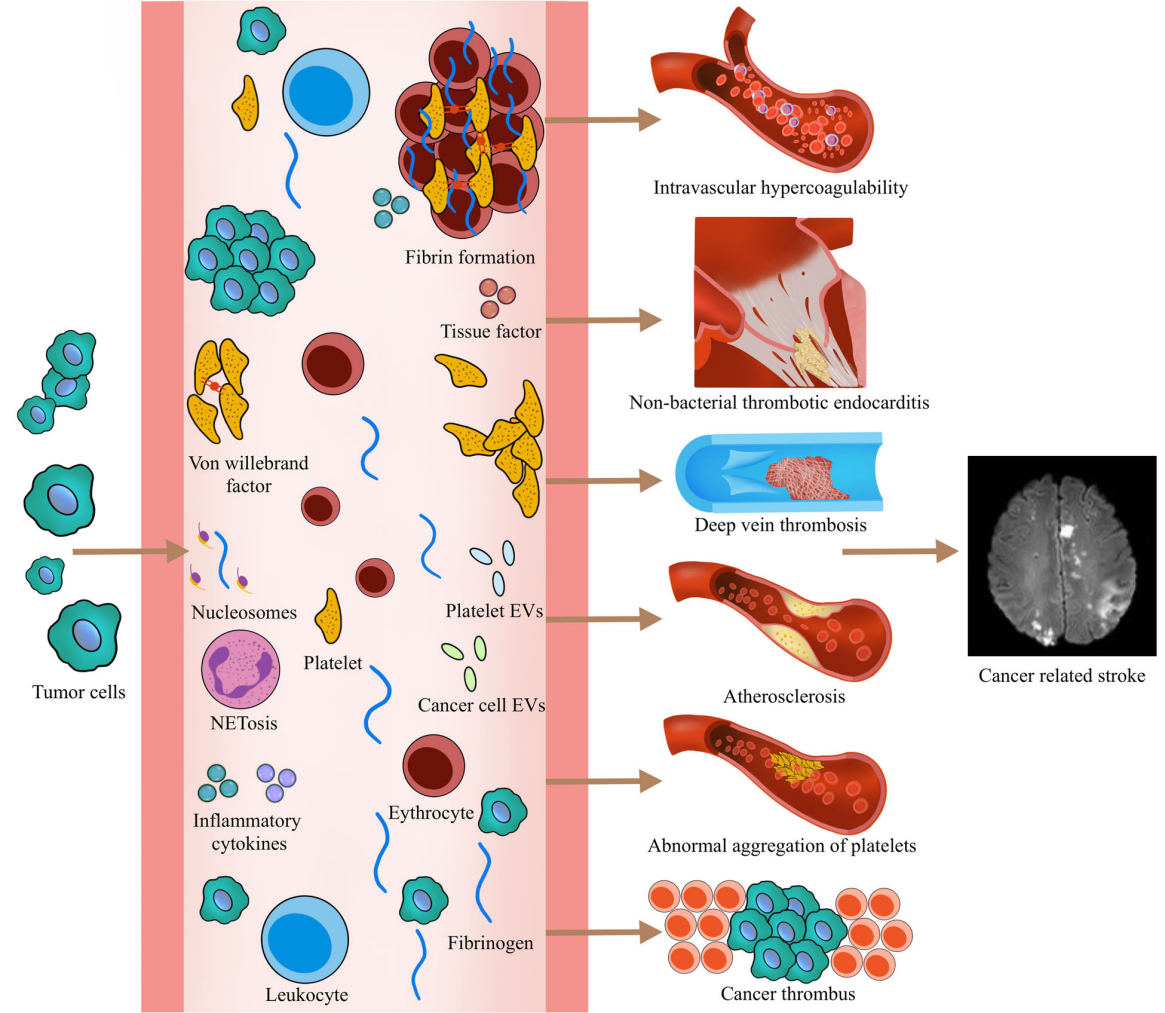
Possible mechanisms between stroke and tumor. EVs, extracellular vesicles; NET, neutrophil extracellular trap

Existing studies on CRS suggest that there are various underlying mechanisms between cancer and stroke (Figure 2). Intravascular hypercoagulability has become the core of the discussion on the pathogenesis of CRS. The center of thrombus formation is thrombin, which is responsible for activating platelets and converting fibrinogen to a fibrin clot. In tumor patients, the level of thrombin is high (Abu Saadeh et al., 2016). Tumor cells increase the potential for thrombin generation both directly, through the expression and release of procoagulant factors, and indirectly, through signals that activate other cell types and components including platelets, leukocytes, erythrocytes, extracellular vesicles (EVs), and neutrophil extracellular traps (NETs) (Reddel et al., 2019). The Vienna Prospective Study of Cancer and Thrombosis (CATS) has found that patients with elevated thrombin levels are at increased risk of cancer‐related thrombosis (Ay et al., 2011).

In CRS patients, elevated plasma DNA levels were found to be associated with CRS by evaluating NET‐specific biomarkers—plasma DNA and nucleosomes—and it was concluded that NETosis is one of the molecular mechanisms of CRS (Bang et al., 2016). This finding was confirmed in pancreatic cancer patients (a cancer type that often accompanies hypercoagulability) (Yu et al., 2020). In the study of the pathological mechanism of stroke in cancer patients, it was found that cancer cells of patients with CRS were able to release higher levels of EVs, and EVs were associated with D‐dimer levels. Further investigation revealed that such EVs were not associated with TF levels (Bang et al., 2019).

Riewald & Ruf (2001) observed that the high expression of tissue factor (TF) is related to cancer‐related thromboembolism. This 47 kDa transmembrane glycoprotein is up‐regulated in cancer cells, and its soluble form also activates the coagulation pathway (Sun et al., 2015). In cancer tissues, TF is expressed in both tumor cells and endothelial cells and is the main inducer of blood coagulation (Hisada & Mackman, 2019). TF can form a complex with factor VII and activate factor VIIa, which further leads to the activation of coagulation factor X and the production of thrombin required for physiological hemostasis. When tumor cells metastasize, the hypercoagulability in the patient can be further activated (L. Liu et al., 2014; Mego et al., 2015).

Higher D‐dimer level is a manifestation of hypercoagulability in cancer patients. It is known that D‐dimer is a direct indicator of blood coagulation function and has been used as a means to assess hypercoagulability (Sun et al., 2015). Compared with stroke patients without a history of tumors, CRS patients have higher level of D‐dimer (Shen et al., 2020). Authors found that the incidence of microembolic signals of transcranial Doppler thrombosis in cancer patients that had cryptogenic strokes is linearly related to the level of D‐dimer (Seok et al., 2010). The D‐dimer assay is most useful in patients with active cancer and stroke because it can determine its cause, predict the risk of stroke recurrence, and help make precise CRS treatment decisions (Ohara et al., 2020).

There are still gaps in the understanding of the mechanisms of CRH, and it is hoped that ongoing or anticipated trials will provide a better understanding of the pathogenesis of CRS. The realization of personalized therapy for CRS should be guided by mechanism‐based diagnostics and molecular targeting.

### 3.2 Non‐bacterial thrombotic endocarditis (NBTE)

NBTE is considered to be the most common source of embolism in CRS (Patel & Elzweig, 2020). It is caused by circulating cytokines that lead to endothelial damage, which in turn initiates the coagulation process. Fibrin and platelets accumulate at the injury, causing thrombus formation (Dearborn et al., 2014; el‐Shami et al., 2007). NBTE is most common in malignant tumors and hypercoagulable states and can occur in 4% of patients with advanced malignancies (Singh et al., 2007; Vlachostergios et al., 2010). Nguyen and DeAngelis (2006) reported that in about 60% of NBTE patients, malignant tumors were found during the autopsy.

### 3.3 Paradoxical embolism

The typical manifestation of cancer hypercoagulability is vascular embolism, most commonly found in the venous system. The risk of venous thromboembolism (VTE) in patients with active cancer is four to eight times that of normal people (Salazar‐Camelo et al., 2021). Áinle & Kevane (2020) and Porfidia et al. (2019) have shown that after VTE, the risk of stroke and other related arterial embolism is greatly increased (. In the early stages of cancer diagnosis and VTE, there is a high risk of suffering from arterial embolism again. In addition, it has also been reported that the embolism of CRS may be related to a patent foramen ovale (PFO). PFOs are present in 25% of the general population, and higher in the cryptogenic stroke population. The putative mechanism of ischemic stroke in patients with PFO is the migration of emboli from the right to the left atrium. A PFO may be the initial manifestation of paradoxical embolism or cancer (Potugari et al., 2019; Tadokoro et al., 2013). In patients with a history of VTE, the emboli can enter the artery through the orifice of the PFO, leading to cerebrovascular embolism.

## 4 MECHANISMS BY WHICH ANTICOAGULATION THERAPY MAY NOT BE EFFECTIVE

In theory, anticoagulation therapy should be effective if we take into account the hypercoagulable mechanism of cerebral infarction in cancer patients. The neutral results of anticoagulation therapy trials indicate that the effectiveness of anticoagulation therapy in the above subgroups is affected by other mechanisms in the CRS population.

### 4.1 Atherosclerosis

Traditional stroke mechanisms, such as atherosclerosis, are a prevalent cause of stroke in cancer (Karlińska et al., 2015; Navi et al., 2014; Y. Zhang et al., 2007). Past autopsy studies have shown that the most common cause of ischemic stroke in cancer patients is still atherosclerosis (Adams, 2019). In addition, cancer‐related treatments, such as radiotherapy, may increase vascular damage and further increase the risk of developing this condition (Chow et al., 2018; Halle et al., 2010). S. G. Kim et al. (2010) found that among patients with traditional stroke risk, the distribution of stroke subtypes of cancer patients in the traditional stroke group was similar to that of stroke patients without cancer. Therefore, it is speculated that tumor‐specific mechanisms are unlikely to play a role in the development of strokes in patients at risk of having a traditional stroke. In a word, after a comprehensive evaluation in CRS patients, a careful vascular examination is necessary to determine the presence or absence of plaque in stroke patients.

### 4.2 Abnormal aggregation of platelets

Previous studies have shown that platelet activation markers are elevated in patients with malignant tumors, including soluble P‐selectin, soluble CD40 ligand, integrin αⅡbβIII, platelet activation receptors, platelet‐derived growth factors, and chemokines (Contursi et al., 2017; Foss et al., 2020; Wojtukiewicz et al., 2017). In patients with thrombosis, tumor‐derived thrombin generation leads to platelet activation through the cleavage of platelet thrombin receptors PAR1and PAR4, and the formation and aggregation of platelets lead to embolism (Tesfamariam, 2016; Q. Zhang et al., 2017). Researches on platelet subgroups (Dale, 2017; Heemskerk et al., 2013; Hua et al., 2015; Kempton et al., 2005) point out that aggregatory platelets contribute to the formation of platelet aggregation through the activation of GPIIb‐IIIa. It was also found that aggregatory platelets participate in the coagulation process to generate the thrombin and that interaction between platelets and thrombin further enhances the blood clotting activity in tumor patients. In addition, activated platelets can make circulating tumor cells escape immune attack and destruction, promoting tumor cell proliferation and metastasis (Bruno et al., 2018; Catani et al., 2020; Patmore et al., 2020). The positive role of platelets in cancer patients provides a theoretical basis for the use of antiplatelet drugs. Therefore, detecting the levels of platelets and platelet activation markers is beneficial for selecting personalized treatment plans for CRS patients.

### 4.3 Cancer thrombus and cancer comorbidities

Tumor ruptures during the growth process, and the thrombus can directly invade the blood vessel, leading to stroke (Bonnet et al., 2015). For these patients, intravascular interventional therapy may be their preferred choice (Byon et al., 2016; Hughes et al., 2015; Uneda et al., 2016). From being diagnosed with cancer, the patient has to undergo surgery, chemotherapy, or radiotherapy and these treatments might increase the risk of thromboembolism. Radiation therapy can cause vascular damage and increase the risk of cardiovascular diseases (Delanian, 2021; van Aken et al., 2021). Related studies have confirmed that the incidence of carotid artery stenosis and ischemic stroke increased after radiotherapy in patients with head and neck tumors (Laugaard Lorenzen et al., 2020; Makita et al., 2020). The effects of long‐term consumption and anti‐cancer treatments make patients immunodeficient, which increases their odds of getting an infection. Patients with a history of infection have a higher risk than those without any episodes in their clinical background.

## 5 MULTIPLE MECHANISMS WORK TOGETHER

The above‐mentioned mechanisms of CRS may not be isolated, and common risk factors might often coexist. In some cases, these coexisting mechanisms may reinforce each other and produce a synergistic effect, leading to thromboembolism. For example, NBTE works through a hypercoagulable system that can lead to embolism. Tumor‐related treatments could cause strokes by enhancing traditional stroke mechanisms. Thrombin can promote the activation and aggregation of platelets and cause vascular embolism.

## 6 CURRENT EXPLORATION OF PERSONALIZED THERAPY STRATEGIES

### 6.1 Secondary prevention of CRS

Currently, there are no relevant regulations on the treatment of CRS based on the evidence of the guidelines. Experimental evidence has shown that the use of anticoagulants in patients with high D‐dimer levels can reduce the incidence of arterial infarction events and VTE events (Castellucci et al., 2013; Oldgren et al., 2013). For example, in the prospective OASIS‐Cancer study, the 1‐year survival rate of cancer and stroke patients whose D‐dimer decreased after anticoagulant treatment was improved (M. Lee et al., 2017). Studies show P‐selectin and L‐selectin mediate coagulation activation. The anticoagulant effect of heparin through the interaction between both is the first choice in the treatment of patients with cancer‐related thromboembolism (Wahrenbrock et al., 2003). Kawano et al. (2019) believe that long‐term subcutaneous heparin treatment may prevent the recurrence of CRS. Current international guidelines all recommend LMWH as the initial and long‐term treatment for cancer‐related venous thrombosis (Farge et al., 2016; Kearon et al., 2016; Streiff et al., 2018). However, since the effectiveness of the treatment depends on the drug being injected subcutaneously, compliance with long‐term treatment is poor. A study conducted in 2017 showed that DOAC and LMWH have similar clinical efficacy and safety in the treatment of cryptogenic ischemic stroke in patients with active cancer (Nam, Kim, Kim, An, Oh, et al., 2017). Factor Xa inhibitors are routinely used in the secondary prevention of atrial fibrillation stroke. However, study of anticoagulant therapy for stroke in atrial fibrillation and CRS has found that they respond differently to anticoagulants (H. Kim et al., 2021). The coagulation mechanism of CRS may be mediated by a factor Xa‐independent pathway. There are many complex and undiscovered mechanisms of CRS; it is necessary to further explore the molecular mechanism of CRS hypercoagulability and determine the best strategies for stroke prevention in cancer patients. Experimental data on secondary prevention comparison of CRS in recent years are summarized in Table 2.

**TABLE 2:** Comparison of secondary prevention strategies for CRS patients

Year of publication	Compared drugs	Test population (n)	Observation time (months)	Results					
				Initial/Recurrence	P value	Major bleeding	P value	Death	P value
2017(Nam, Kim, Kim, An, Oh, et al., 2017)	LMWH vs. NOAC	41 vs. 7	3	49.0% vs. 57.0%	0.846	39.0% vs. 29.0%	0.696	59.0% vs. 57.0%	1.000
2018(Navi, Marshall, et al., 2018)	Enoxaparin vs. Aspirin	10 vs. 10	12	5.0% vs. 7.0%	0.302	10.0% vs. 30.0%	0.582	N/A	N/A
2020(Martinez‐Majander et al., 2020)	Rivaroxaban vs. Aspirin	254 vs. 289	11	7.7% vs. 5.4%	0.275	2.9％ vs. 1.1％	0.950	3.7% vs. 3.3	0.780
2021(Yamaura et al., 2021)	UFH vs. DiXals	24 vs. 29	1	4.0% vs. 31.0%	0.015	4.0% vs. 10.0%	0.617	17.0% vs. 17.0%	1.000

Abbreviations: LMWH, low‐molecular weight heparin; NOAC, new oral anticoagulant; UFH: unfractionated heparin; DiXal: direct factor Xa inhibitor.

Several randomized trials have shown that oral factor Xa inhibitors are equivalent to subcutaneous LMWH in terms of safety and efficacy in preventing recurrent VTE or massive bleeding in cancer patients, making them an attractive choice for cancer‐related ESUS(Agnelli et al., 2020; Carrier et al., 2019; Khorana et al., 2019; Raskob et al., 2018). Also, the latest guidelines recommend that DOAC be used for long‐term maintenance treatment of certain cancer patients to prevent VTE (Key et al., 2020). Since the occurrence of CRS may be more than a mechanism of hypercoagulability, the relevant treatment options for venous thrombosis can be used for reference, but it cannot be directly transferred.

For embolism caused by NBTE, anticoagulation therapy is still the basis. Current guidelines recommend heparin as the first‐line treatment for NBTE (Whitlock et al., 2012). In addition, for patients with large vegetations, valve dysfunction, or repeated embolism, if anticoagulation therapy is ineffective, valve repair or valve replacement can also be performed (Habib, 2015; J. Liu & Frishman, 2016).

In the TEACH Pilot Randomized Clinical Trial, Navi, Marshall, et al. (2018) tried to compare the effects of aspirin and heparin in treating malignant tumors with cerebral infarction. After 1‐year follow‐up, they have found no significant difference in the cumulative incidence of significant bleeding, thromboembolic events, and survival rates between the two groups of patients (*p* > 0.05). The subgroup analysis of the NAVIGATE ESUS randomized trial (Martinez‐Majander et al., 2020) found that patients with and without a history of cancer had a similar incidence of recurrent ischemic stroke and all‐cause mortality during aspirin and rivaroxaban treatment. It was stated that aspirin was safer than rivaroxaban for significant bleeding. Considering the above test results, we can conclude that for the secondary prevention of CRS patients, anticoagulation therapy should be feasible, but it has not shown evident benefits as we expected. More clinical trials are needed to confirm the best anticoagulant therapy for patients in these conditions.

When it comes to anticoagulant therapy for cancer patients, we must further discuss the risk of bleeding. A risk of bleeding may be even higher in cancer patients with stroke and other brain pathologies (Kamphuisen & Beyer‐Westendorf, 2014; Mantia et al., 2017). Major bleeding in cancer patients is associated with an increased risk of death, with the highest rates of major bleeding found in the elderly and people with medical comorbidities, gastrointestinal or urogenital cancers, and metastatic diseases, which are common in cancer‐related ESUS (A. Lee, 2017).

There are many potential causes of ESUS; both white thrombus and red thrombus are involved in the process of thrombus formation. Therefore, some scholars have hypothesized that the combined use of anticoagulants and antiplatelet drugs in ESUS patients can significantly reduce the overall burden of thrombosis, thereby reducing the risk of recurrent stroke. This hypothesis is supported by results from COMPASS (Cardiovascular Outcomes in People Using Anticoagulation Strategies), in which the combination of low‐dose rivaroxaban and aspirin was associated with a substantially lower risk of stroke compared with aspirin as monotherapy (Eikelboom et al., 2017; Sharma et al., 2019). Therefore, we speculate whether antiplatelet combined with anticoagulation strategies will be suitable for secondary prevention strategies of CRS, which requires clinical trials to verify.

In the traditional secondary prevention strategies for stroke, besides anti‐platelet aggregation, statins are also used. The paper of Boulet aimed to explore whether statins reduce radiation‐induced vascular complications in cancer patients postradiotherapy to the thorax, head, and neck. The results showed that statin use post radiation therapy was associated with a significant reduction in stroke, with a trend toward significantly reducing cardiovascular and cerebrovascular events (Boulet et al., 2019). Therefore, the use of statins in patients with a history of radiation therapy may be effective in preventing stroke risk.

### 6.2 Treatment of acute ischemic stroke in CRS

Many scholars have discussed whether thrombolysis is suitable for patients with malignant tumours associated with episodes of acute stroke, but the conclusions are not consistent. Murthy et al. (2013) observed 32,576 stroke patients, who received thrombolytic therapy. After adjusting for confounding factors, there was no significant difference in discharge rate and in‐hospital mortality in patients with malignant tumour compared with those with non‐malignant clinical cases. Still, the incidence of all cerebral hemorrhages in both groups was similar. However, the multivariate analysis presented in a retrospective study of 13,993 patients with acute ischemic stroke treated by thrombolysis revealed that the final mortality rate of patients with malignant tumours did not significantly increase compared with other patients, but the incidence of bleeding was higher (Weeda & Bohm, 2019). In another large clinical study, it was found that the presence or absence of a history of cancer had no significant effect on intracranial hemorrhage, all‐cause hemorrhage, or hospital mortality after thrombolytic therapy (Owusu‐Guha et al., 2021).

The results of the above data confirm the position of thrombolytic therapy in patients with CRS, but bleeding after thrombolysis is inevitable, and patients with primary or metastatic central nervous system malignant tumours may increase the risk of post‐thrombolysis bleeding due to vascular changes or damage (Fugate & Rabinstein, 2015; Masrur et al., 2011). Therefore, it is inferred that the deviation of the bleeding risk in the above experimental results may be due to the inconsistent number of patients with metastatic cancer among the groups. A retrospective study showed that if a cancer patient concurrently metastasizes to other sites, especially stomach, esophagus, hepatocellular carcinoma, or pancreatic cancer, the survival time will be greatly shortened; even if reperfusion treatment is performed, 80% to 100% of people die within 6 months after stroke (Yoo et al., 2019).

At present, mechanical thrombectomy is considered a first‐line treatment method for acute anterior circulating large vessel occlusive stroke (Berkhemer et al., 2015; Campbell et al., 2015; Goyal et al., 2015; Jovin et al., 2015; Saver et al., 2015). Several trials have explored the applicability of thrombectomy for patients diagnosed with tumours (refer to Table 3). After analyzing the results of these trials, we propose that when acute cerebral infarction occurs in such patients, immediate thrombectomy therapy should be feasible. However, for patients with later‐stage tumors, a more careful approach is necessary since they may have a poor prognosis.

**TABLE 3:** Treatment of acute ischemic stroke in CRS‐ER

Year of publication	Test population (n)	TICI 2b or 3	P value	ICH	P value	mRS 0−2 at 3 months	P value	Death at 3 months	P value	Intrahospital mortality	*p* value
2018(Jung et al., 2018)	CRS (19) vs. LAA (105) vs. CE (205)	63.0% vs. 84.0% vs. 84.0%	0.060	N/A	N/A	16.0% vs. 54.0% vs. 44.0%	0.008	63.0% vs. 4.0% vs. 13.0%	< 0.001	N/A	N/A
2019(Sallustio et al., 2019)	CRS (24) vs. Non‐cancer (24)	76.9% vs. 61.5%	0.670	25.0% vs. 29.0%	1.000	25.0% vs. 29.1%	1.000	29.1% vs. 12.5%	0.280	8.3% vs. 4.1%	1.000
2019(D. Lee et al., 2019)	CRS (26) vs. Non‐cancer (227)	88.5% vs. 90.7%	0.723	57.7% vs. 38.7%	0.034	23.1% vs. 41.9%	0.064	30.8% vs. 8.8%	0.003	N/A	N/A
2020(Cho et al., 2020)	CRS (27) vs. Non‐cancer (351)	85.2% vs. 82.6%	0.800	44.4% vs. 32.8%	0.290	37.0% vs. 39.6%	0.840	33.3% vs. 8.2%	< 0.001	3.7% vs. 2.3%	0.490
2021(E. Lee et al., 2021)	CRS (34) vs. Non‐cancer (307)	79.4% vs. 86.7%	0.103	41.2% vs. 23.8%	0.037	N/A	N/A	26.5% vs. 6.8%	< 0.001	20.6% vs. 5.9%	0.009
2021(Ciolli et al., 2021)	CRS (14) vs. Non‐cancer (267)	71.0% vs. 78.0%	0.520	43.0% vs. 40.0%	1.000	21.0% vs. 44.0%	0.160	64.0% vs. 14.0%	< 0.010	43.0% vs. 6.0%	< 0.010

Abbreviations: ERT, endovascular recanalization therapy; ICH, intracranial hemorrhage.

## 7 CONCLUSIONS AND PROSPECTS

With the aging of the world population and the prolonged survival time of cancer patients, the incidence of CRS is gradually increasing and it is expected that CRS will become a common subtype of traditional stroke. Regarding CRS as a subtype of traditional stroke is conducive to the establishment of a standardized diagnosis and treatment system for such patients. Although this definition does not yet guide treatment, it is the basis for further diagnosis and can help clinicians adopt more effective and tailored therapy strategies to prevent strokes. At present, the pathogenesis of CRS is still not unclouded, the existing secondary stroke prevention and acute treatment programs for such patients are still in the exploratory stage. This article reviews the current possible mechanisms for the pathogenesis of CRS and the corresponding exploratory treatment options based on these pathogeneses. It is hoped that this article can contribute to the formulation of personalized therapy plans for CRS.

## CONFLICT OF INTEREST

The authors declare that they have no conflicts of interest.

## AUTHOR CONTRIBUTIONS

Yu‐Jie Chen, Rui‐Guo Dong, and Jie Sun conceived and designed the study, analyzed the data, interpreted the study findings, and drafted the manuscript. Jie Sun conceived and designed the study. Meng‐Meng Zhang analyzed data. Chao Sheng and Peng‐Fei Guo supervised and directed the conduct of the study. Yu‐Jie Chen critically reviewed the manuscript. All authors had full access to all the data and the accuracy of the data analysis. The authors read and approved the final manuscript.

### PEER REVIEW

The peer review history for this article is available https://publons.com/publon/10.1002/brb3.2738.
